# Supplementary material for: Sequence and Structure Properties Uncover the Natural Classification of Protein Complexes Formed by Intrinsically Disordered Proteins via Mutual Synergistic Folding
Source: Int J Mol Sci. 2019 Nov 1;20(21):5460. doi: 10.3390/ijms20215460 (PMC6862064; doi:10.3390/ijms20215460)
Supplement: Supplementary file 1 [file ijms-20-05460-s001.zip › ijms-625444-supplementary materials-proofreading/Supplementary Figures S1-S4.docx]

Supplementary Material


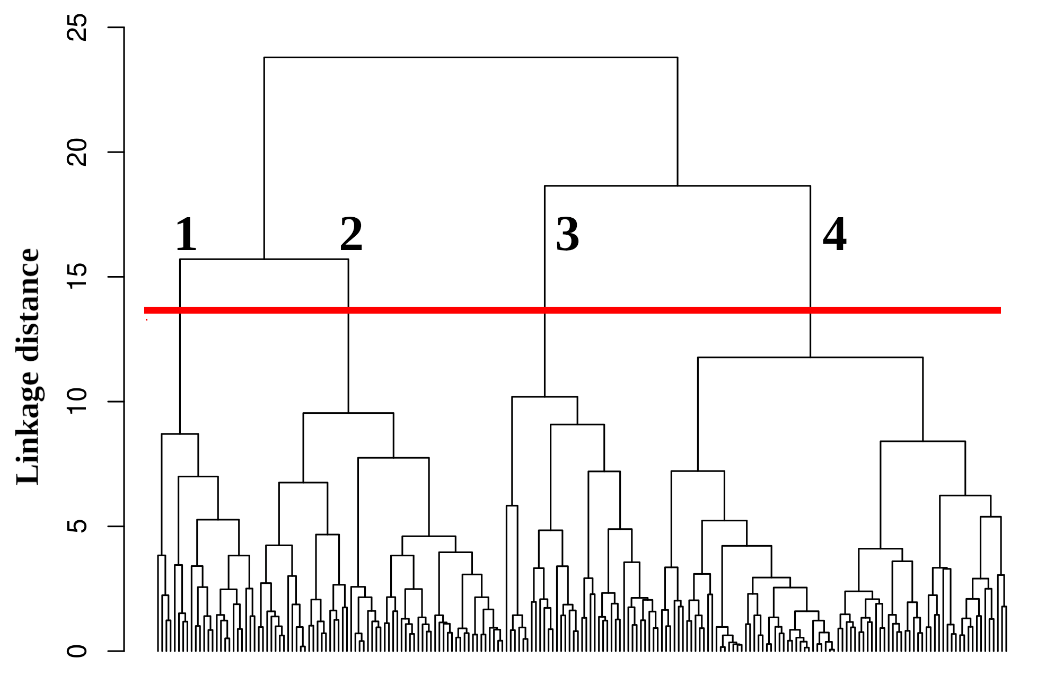


**Figure S1.** Sequence-based hierarchical clustering of complexes formed via MSF. Red line marks the cutoff used to define the four sequence-based clusters.


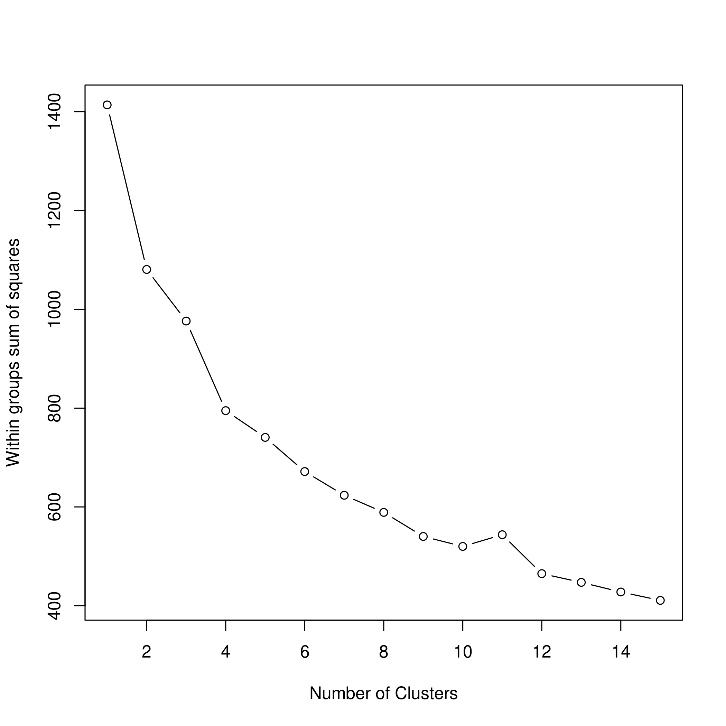


**Figure S2.** The within groups sum of squares as a function of the number of clusters. k-means clustering was done using sequence parameters as input.


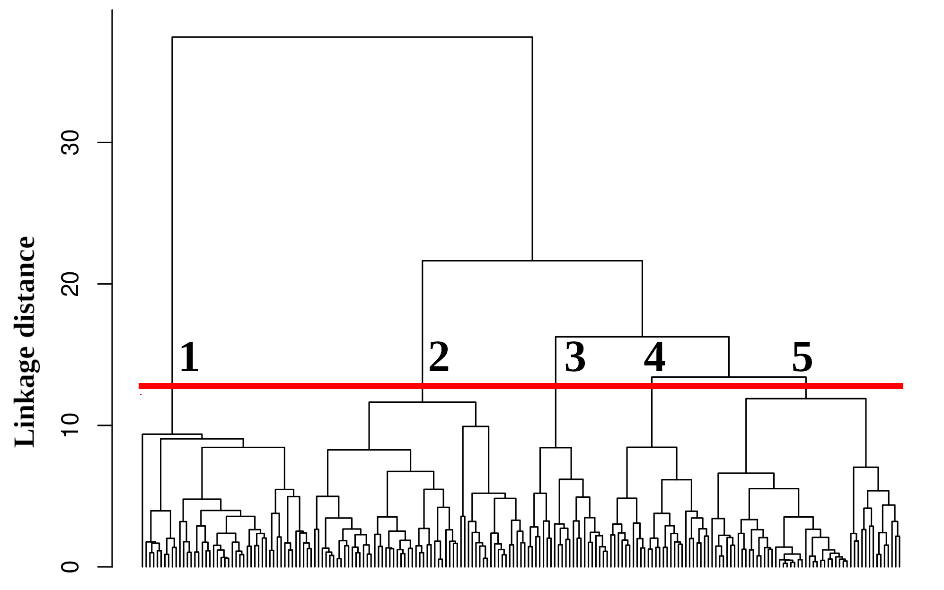


**Figure S3.** Structure-based hierarchical clustering of complexes formed via MSF. Red line marks the cutoff used to define the five structure-based clusters.


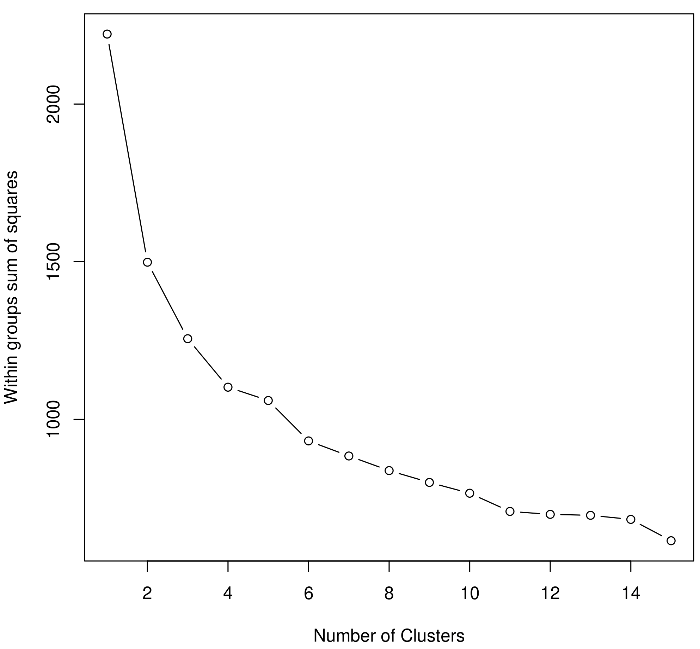


**Figure S4.** The within groups sum of squares as a function of the number of clusters. k-means clustering was done using structure parameters as input.
